# Supplementary material for: The causal relationship between the human gut microbiota and pyogenic arthritis: a Mendelian randomization study
Source: Front Cell Infect Microbiol. 2024 Nov 26;14:1452480. doi: 10.3389/fcimb.2024.1452480 (PMC11629706; doi:10.3389/fcimb.2024.1452480)
Supplement: Supplementary file 1 [file DataSheet1.zip › mendelian test/ebi-a-GCST90027626.csv_forest.pdf]

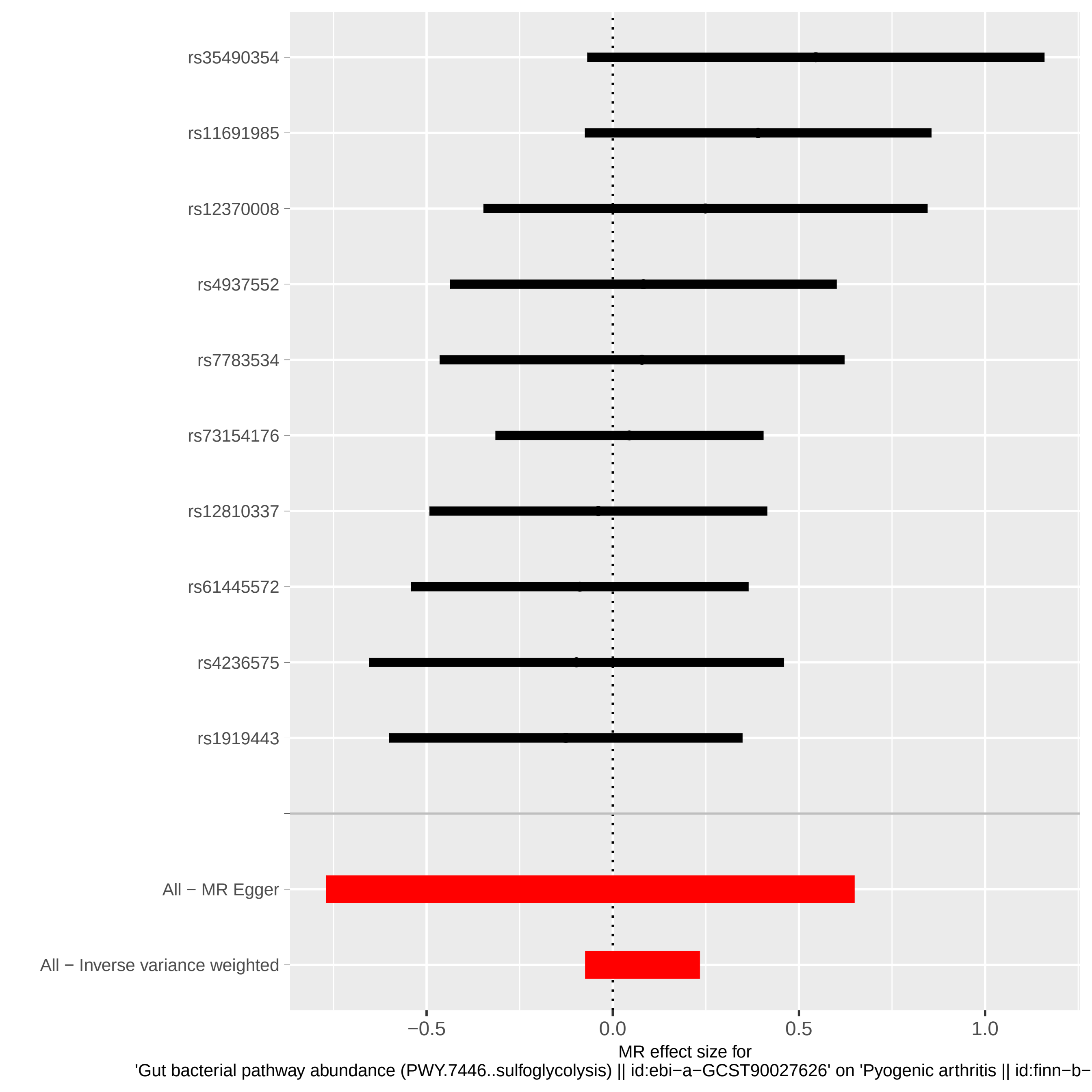

rs35490354
rs11691985
rs12370008
rs4937552
rs7783534
rs73154176
rs12810337
rs61445572
rs4236575
rs1919443
All − MR Egger
All − Inverse variance weighted
−0.5
0.0
0.5
1.0
MR effect size for
'Gut bacterial pathway abundance (PWY.7446..sulfoglycolysis) || id:ebi−a−GCST90027626' on 'Pyogenic arthritis || id:finn−b−
